# *Bacillus megaterium* WL-3 Lipopeptides Collaborate Against *Phytophthora infestans* to Control Potato Late Blight and Promote Potato Plant Growth

**DOI:** 10.3389/fmicb.2020.01602

**Published:** 2020-07-09

**Authors:** Youyou Wang, Jiao Liang, Congying Zhang, Le Wang, Wenbin Gao, Jizhi Jiang

**Affiliations:** College of Life Science, Institute of Life Sciences and Green Development, Hebei University, Baoding, China

**Keywords:** *Bacillus megaterium*, *Phytophthora infestans*, lipopeptides, potato late blight, promotional growth

## Abstract

Oomycete *Phytophthora infestans* [(Mont.) de Bary] is the cause of potato late blight, a plant disease which poses a serious threat to our global food security and is responsible for huge economic losses worldwide. Lipopeptides produced by *Bacillus* species are known to be potent antibacterial compounds against many plant pathogens. In this study, we show that *Bacillus megaterium* WL-3 has an antagonistic effect against potato late blight. Electrospray ionization mass spectrometry (ESI-MS) revealed that lipopeptides derived from the WL-3 strain contained three subfamilies, surfactin (C_13_ – C_15_), Iturin A (C_14_ – C_16_), and Fengycin A (C_15_ – C_19_). The Iturin A and Fengycin A lipopeptide families were each confirmed to have anti-oomycete effects against *P. infestans* mycelium growth as well as obvious controlling effects against potato late blight in greenhouse experiments and field assays. Furthermore, Iturin A and Fengycin A were able to promote plant photosynthetic efficiency, plant growth, and potato yield. Most importantly, the combination of Iturin A and Fengycin A (I + F) was superior to individual lipopeptides in controlling potato late blight and in the promotion of plant growth. The results of this study indicate that *B. megaterium* WL-3 and its lipopeptides are potential candidates for the control of late blight and the promotion of potato plant growth.

## Introduction

Potato (*Solanum tuberosum* L.) is a perennial herbaceous plant of the Solanaceae family, the fourth most produced food crop in the world, directly behind maize, wheat, and rice, respectively (Rajiv and Kawar, 2016; Wan, 2017). It will soon replace maize and become the third largest staple food in China (Lu, 2015). However, potato production is often disrupted by a variety of plant pathogens, of which late blight, caused by the oomycete *Phytophthora infestans* [(Mont.) de Bary], is the most destructive and intractable disease (Schepers et al., 2018). At present, except for a selection of disease-resistant varieties, potato crops are predominantly treated with chemicals in order to control the development of late blight (Caulier et al., 2018; Wang, 2018). The excessive use of chemical pesticides has resulted in increasingly serious environmental pollution and has accelerated the physiological differentiation and variation of *P. infestans* (Jin et al., 2017; Fukue et al., 2018). As a result, the highly pathogenic A2 mating type appears in many potato-producing areas, and its frequency has increased year by year (Wan, 2017). Currently of greatest concern is the frequent appearance of full-spectrum physiological races capable of overcoming the resistance genes *R1* to *R11* (Jin et al., 2017; Fukue et al., 2018). In recent years, increasing attention has been given to biocontrol measures, which involve such mechanisms as induced systemic resistance (ISR), competition, antagonism, and plant growth promotion (Li et al., 2014). Biocontrol methods, primarily the use of *Bacillus* species or their metabolites to control plant diseases, have become a focus of phytopathogen research (Meena and Kanwar, 2015).

*Bacillus* strains can produce numerous antibiotics, such as cyclic lipopeptides (CLPs), which have gained attention because of their wide range of antagonistic activities and high-yield of production (Ongena and Jacques, 2008). Three main subfamilies of CLPs, the surfactins, iturins, and fengycins, have fatty acid chains of different lengths and numbers of amino acids (Ongena and Jacques, 2008; Perez et al., 2017). According to previous reports, CLPs produced by different antagonistic strains differ greatly in their activities. Among those CLPs, surfactins exhibit marked antibacterial activities (Ongena and Jacques, 2008). The iturins and fengycins have activities against fungal pathogens such as *Candida albicans*, *Sclerotinia sclerotiorum*, *Botrytis cinerea*, *Fusarium graminearum*, and *Magnaporthe grisea* (Moyne et al., 2001; Romero et al., 2007; Kumar et al., 2012; Tabbene et al., 2015; Zhang and Sun, 2018). However, no studies as yet have determined or compared the individual contributions of particular CLPs such as surfactins, fengycins, and iturins against the oomycete *P. infestans* (Gong et al., 2015). In addition, synergism between CLPs to control potato late blight is still unknown (Wang, 2018; Zhang and Sun, 2018), and it is also not known how changes in environmental factors, such as temperature, humidity, and microorganism interference might affect the antagonistic effects (Ongena et al., 2005; Parthipan et al., 2017). Therefore, it is worth investigating CLPs for their biocontrol effect against late blight in field trials. Some reports have suggested that CLPs induce resistance in plants as their antagonistic mechanism against plant pathogens (Wang, 2018). In addition, the intrinsic promotion of potato plant growth by CLPs is also a worthwhile aspect of protection to investigate (Perez et al., 2017).

In this study, the CLPs extracted from the *B. megaterium* WL-3 strain were classified using mass spectrometry (MS) and their specific antagonistic activities against *P. infestans* were determined. The biocontrol effects of two CLPs subfamilies, Iturin A and Fengycin A, against late blight in both greenhouse and field trials were assessed, and CLPs promotion of potato plant growth was also evaluated. The results of these studies suggest that the *B. megaterium* WL-3 strain and its CLPs, Iturin A and Fengycin A, are potential anti-oomycete agents for the control of potato late blight.

## Materials and Methods

### *Bacillus megaterium* WL-3 Against *Phytophthora infestans*

*Phytophthora infestans* [(Mont.) de Bary] W101 was obtained from China General Microbiological Culture Collection Center (CGMCC 3.19919) and cultured on Rye (R) solid medium at 20°C (Wang et al., 2017). *B. megaterium* WL-3 (MK241789) was isolated from *Capsicum frutescens* leaves and cultured on Luria Bertani (LB) solid medium at 35°C (Wang, 2018). *P. infestans* mycelium disk (diameter = 7 mm) was applied to the R solid medium (diameter = 9 cm) and cultured for 3 days, at which time the prepared *B. megaterium* WL-3 Living cell (LC), Cell suspension (CS, 1 × 10^7^ CFU/mL, 100 μL), and Cell-free supernatant (CFS, 100 μL) were evaluated for their inhibitory effect using the plate dual culture method (Kunova et al., 2016). LB medium was used as the control. After 5 days incubation at 20°C, the inhibition rates were calculated according to the following formula (Ding et al., 2017):

Inhibitionrate(%)=(C-T)/C×100

Where, C represents the oomycete colony radius of the control, and T represents the radius of the treatment groups. The experiments were performed three times (experimental replication) and in triplicate three groups were parallel (technical replication). Data are expressed as the average ± standard deviation.

### *B. megaterium* WL-3 Biocontrol Assays on Potato Tissues *in vitro*

*Phytophthora infestans* mycelium was collected and oscillated to expose sporangium (1 × 10^7^ CFU/mL), and zoospores (1 × 10^7^ CFU/mL) were collected after release at 10°C for 3 h (Wang, 2018). The sensitive variety of potato, “Bintje” plant (60 days), was used to produce tubers (2.0 cm × 2.0 cm × 0.5 cm) and leaflets for *in vitro* experiments (Jiang et al., 2017). Biocontrol experiments were conducted in three scenarios as follows: Disease prevention (DP): CS (20 μL) was applied to the tuber and leaflet upper surfaces at 20°C in the dark for 48 h in advance, then, the zoospore suspension (20 μL) was applied to the back surface of the leaflets, and a *P. infestans* mycelium disk (diameter = 7 mm) was placed on the upper surface of tubers. Disease index (DI) was determined after 7 days incubation at 20°C (Jiang et al., 2017). Simultaneous inoculation (SI): The CS and *P. infestans* (zoospore suspension/mycelium disk) inoculation method was the same as described for the DP treatment; however, the CS and *P. infestans* were applied at the same time. Disease therapy (DT): The same above, infection of *P. infestans* was carried out for 48 h prior to application of CS. Equal volume of LB liquid medium and the application of CS only without infection were considered as the controls. After 7 days incubation at 20°C, DI was calculated based on the rating scale of 0 – 9. Where, 0 = no symptoms; 1 = less than one third of the total leaflet/tuber with symptoms; 3 = one third to half of leaflet/tuber with symptoms; 5 = one half to two thirds leaflet/tuber with symptoms; 7 = more than two thirds leaflet/tuber with symptoms; 9 = all leaflet/tuber with symptoms (Han et al., 2016; Guo et al., 2020). The DIs were calculated by the following formula (Balint-Kurti et al., 2007; Ding et al., 2017):

DI=Σ(d×il)i/(L×N)×100

where, d_*i*_ represents the grade of disease severity, and l_*i*_ is defined as the number of leaflets or tubers at different grades of disease. L is the number of samples investigated, and N represents the highest grade 9 of disease severity. The experiments were performed three times (experimental replication, total 36 samples in each treatment) with triplicates in each experiment (technical replication). Data are expressed as the average (36 samples) ± standard deviation.

### *B. megaterium* WL-3 Crude Lipopeptides Extract (CLE) and Disk Diffusion Assay

CS (1 × 10^7^ CFU/mL) of *B. megaterium* WL-3 strain (60 mL/L) was transferred into 50 L Landy fermentation medium (30°C, 170 rpm) and cultured for 96 h to accumulate CLPs (Yang et al., 2015). According to the acid precipitation method (Medeot et al., 2017), fermentation medium was centrifuged (1 × 10^4^ rpm, 4°C, 10 min) to remove LC. The supernatant was precipitated using 6 M HCl, and the precipitation was washed with methanol and dried in a rotary evaporator (YARONG, RE52CS-1, Shanghai, China) to obtain CLE for further analysis (Nair et al., 2016). To detect the inhibitory effect of CLE on *P. infestans* mycelium growth, a disk diffusion assay was adopted (Medeot et al., 2017). A *P. infestans* disk (7 mm) was incubated for 3 days, then, CLE water solution (1 mg/mL) was prepared and applied to a filter paper (diameter = 5 mm, 5 μL per paper) on the *P. infestans* plate. An equal volume of distilled water was similarly applied as the control. After 5 days co-incubation at 20°C, the zones of inhibition were measured. Disk diffusion assays were performed three times (experimental replication), with triplicates for each condition in each experiment (technical replication). Data are expressed as the average ± standard deviation.

### MALDI-TOF-MS Analysis of CLE

Crude lipopeptides extract methanol solution (100 μg/mL) was mixed with saturate matrix solution of α-cyano-4-hydroxy-cinnamic acid (1:1, v/v). The matrix solutions were prepared in 1:1 (v/v, CH_3_CN:H_2_O) containing 0.1% trifluoroacetic acid (TFA) (Medeot et al., 2017). MALDI-TOF-MS (MALDI-TOF, AUTOFLEX III, Bruker Daltonics) was operated at an initial accelerating voltage of 20 kV in positive reflective mode to determine the classification of CLE, and the molecular weights at *m/z* 600 – 1,700 were determined (Yang et al., 2015).

### Lipopeptides Purification Using HPLC and MS/MS Analysis

Crude lipopeptides extract methanol solution (100 μg/mL) added with 0.1% TFA was analyzed by high-performance liquid chromatography (HPLC) (Waters-E2695, United States, C_18_ Diamonsil sum, 250 mm × 4.6 mm, 5 μm) system (Farzaneh et al., 2016). Samples were injected into the HPLC system (25°C, 140 bar) in the volume of 1 mL per time, and detected in the wavelength of 214 nm. The flowing system consists of mobile phase A (acetonitrile) and mobile phase B (water) was eluted using the following gradient (%, A:B, v/v): injection start (10:90), 5 min isocratic (10:90), then (35:65) with an increasing gradient of solvent A to 65% through 10 min, next, 5 min isocratic (65:35) followed by 5 min isocratic (80:20), finally, an increasing gradient of solvent A to 100% through 10 min (Medeot et al., 2017). The main peaks (22.3, 24.4, and 25.7 min) after HPLC isolation were collected for MALDI-TOF-MS/MS analysis. The same above, MS/MS (MALDI-TOF, AUTOFLEX III, Bruker Daltonics) system was used in HCD mode to clarify the specific CLPs (1 μg/mL) molecular structure, and the collision energy from 35 to 50 eV was optimized for each precursor ion (Gong et al., 2015).

### Evaluation of Purified Lipopeptide Inhibitory Activities

A disk diffusion assay (5 μL of per paper disk) was used to determine the inhibitory activity of purified CLPs at concentrations of 10, 15, 20, and 25 μg/mL, respectively, and the zones of inhibition were recorded. In addition, the *in vitro* biocontrol experiments of Iturin A and Fengycin A on leaflets (total 36 samples in each treatment) were conducted according to infection/treatment scenarios DP and DT. The protective CLPs were prepared in three groups, Iturin A and Fengycin A individually (25 μg/mL each) and the combination of Iturin A with Fengycin A (I + F, 25 μg/mL each, 1:1 v/v). Equal volumes of Metalaxyl aqueous solution (10 μg/mL) and distilled water were used as positive and negative controls, respectively. After 7 days incubation, the DI was calculated as above. The evaluations of purified lipopeptide inhibitory activity were performed in three independent experiments (experimental replication) with parallel triplicates in each experiment (technical replication). Data are expressed as the average ± standard deviation.

### Greenhouse Experiments

#### Cultivation of Potato Plants and Drug Spraying

The inhibitory effect of Iturin A and Fengycin A on potato late blight was analyzed in a greenhouse with living cycle conditions set at 22°C/15°C (day/night), 70% relative humidity, and 16 h light/8 h dark photocycle (Song et al., 2017). Potato tuber seeds (“Bintje”) were planted three per pot (diameter = 30 cm) containing 500 g nursery substrates (peat:vermiculite:perlite = 3:2:3, v/v), and the plants were irrigated (500 mL/plot) weekly (Cordeiro et al., 2018). Potato plants grown for 30 days were used to detect the inhibitory effects of Iturin A and Fengycin A against late blight. Iturin A, Fengycin A, and I + F were dissolved in sterile water (25 μg/mL, individually, and ratio of 1:1 (in volume) for the combination). Metalaxyl aqueous solution (10 μg/mL) and sterile water were used as the positive and negative controls, respectively. Potato plants were sprayed at 10-day intervals (total three applications) with drug or control (2 mL per pot, 10 pots per treatment) in the biocontrol experiment (30 plants per treatment). The greenhouse experiments were performed three times (experimental replication). Data are expressed as the average ± standard deviation.

#### Biocontrol Effect of Iturin A and Fengycin A in Greenhouse Experiments

Twenty-four hours after the first drug spray, the plants were sprayed (30 day-old plants; 10 pots per group) with the prepared *P. infestans* zoospore suspension (1 × 10^7^ CFU/mL, 1 mL per pot), and placed in a growth chamber with relative humidity at 90% for 24 h to enable infection before returning it to 70% (Clinckemaillie et al., 2016). Disease severity was recorded according to the 1 – 9 scale on days 7, 14, 21, and 28 after the first spraying, and three pots in each group were selected and scored randomly to reduce experimental errors (Balint-Kurti et al., 2007). The DI calculation was the same as above, and the disease reduction (DR) was calculated as follows (Ding et al., 2017):

Disease⁢reduction⁢(DR)=(C-T)×100%

DI of the control and treatment groups were represented by the letters C and T, respectively.

#### Effects on Potato Plant Growth and per Plant Yield in Greenhouse Experiments

The number of potato plants, method of cultivation, and preparation of CLPs (Iturin A, Fengycin A, and I + F) were the same as above (biocontrol experiment). Five days after the tuber seeds had sprouted from the soil, the drugs (Iturin A, Fengycin A, and I + F) were sprayed (2 mL/pot) at 5-day intervals for a total of six applications. The control group was sprayed with equal volumes of distilled water. Finally, the potato plants (35 days-old) were harvested to analyze the growth status including the plant height (PH), main stem diameter (MSD), and main roots number (MRN) (Wang, 2018; Cheng et al., 2019). In addition, after 80 days of growth, plants within each treatment group were harvested to record the average per plant yield (PPY) (Caulier et al., 2018).

### Field Trials

#### Control of Late Blight in Field Trials

Field trials were performed at Baoding University (Hebei, China) test field from May 1 to August 1, 2019. The environmental conditions during this period were suitable for late blight infection and development. A randomized complete block assay was designed with five plots (six rows total 36 hills per replicate, three replicates totaling 108 hills per plot) including Iturin A, Fengycin A, I + F, Metalaxyl suspensions (10 μg/mL), and control (distilled water) groups to cultivate “Bintje” potato plants (total 540 hills in field trials) (Caulier et al., 2018). CLPs and Metalaxyl suspensions were prepared as described above in the greenhouse experiments and sprayed at 1 L per plot (total five plots) every 10 days from May 20 to July 21, 2019 (total seven applications). Disease severity of potato plants selected randomly was monitored and scored for all leaflets (10 hills/plot) every 10 days from May 27 to July 28, 2019 (seven times), and the DI and DR were calculated. In the field experiments, each treatment group was performed in triplicate and in parallel (technical replication), and data are expressed as the average ± standard deviation.

#### Photosynthetic Efficiency and Potato Yield

The potato plant cultivation methods, CLPs solution (Iturin A, Fengycin A, and I + F) preparations, and spraying process were the same as described above (field trials). The control group was sprayed with equal volumes of distilled water. The photosynthetic efficiency was measured using a portable photosynthetic apparatus (LI-6800F, Licor, Lincoln, NE, United States), and the leaflet chamber area was 6 cm^2^ (Chen, 2017). Photosynthetic rate (*P*_*N*_), stomatal conductance (*g*_*s*_), and transpiration rate (*E*) were determined to evaluate photosynthetic efficiency, and the intrinsic water use efficiency (*WUE*_*i*_) was defined as the ratio of *P*_*N*_ to *g*_*s*_ (Cao, 2015). The upper parts of healthy leaflets (three leaflets/hills, six hills) were harvested each day on days 3 – 5 (three consecutive days) after the first, third, and fifth spraying in order to measure the photosynthetic efficiency (nine records) and the final results were based on the average of nine records. Once plants reached physiological maturity (∼90 days), they were harvested to record the potato number and yield of per hill (plant) (Caulier et al., 2018).

### Statistical Analysis

The statistically significant difference between different groups was determined using SPSS software (version 22, IBM, United States) and one-way analysis of variance (ANOVA). *P-*values < 0.05 were considered statistically significant.

## Results

### *B. megaterium* WL-3 Activity Against Mycelium Growth and Biocontrol Effect on Potato Plant Tissues *in vitro*

**FIGURE 1:**
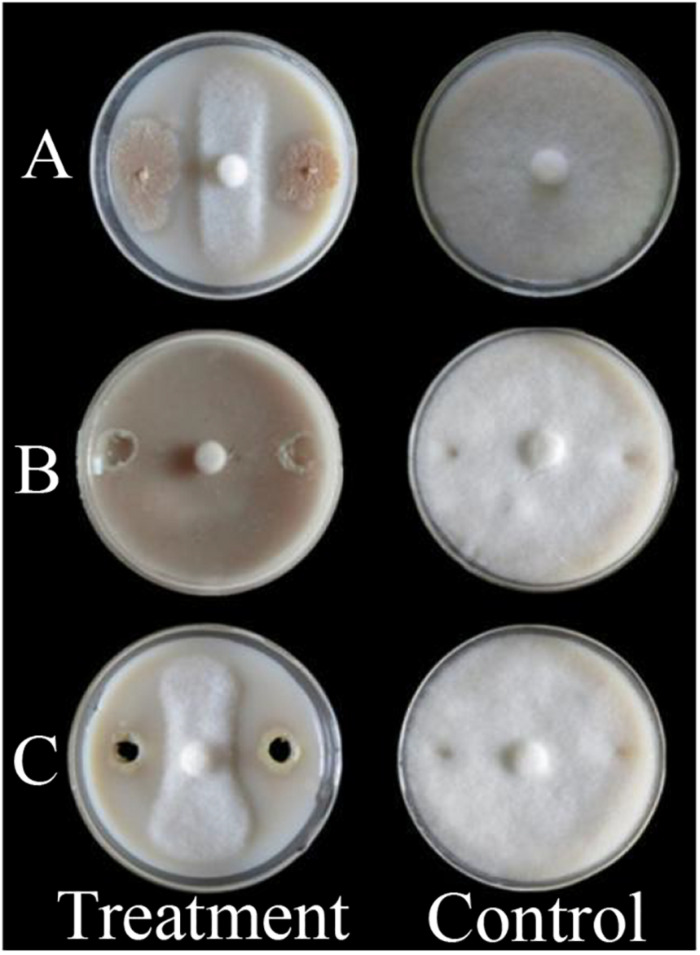
Inhibitory effect of *B. megaterium* WL-3 against *P. infestans* mycelium growth. **(A)** Living cell (LC); **(B)** Cell suspension (CS); **(C)** Cell-free supernatant (CFS). Photographs are representative of experiments performed in triplicate, the same below.

Obvious inhibitory effects of *B. megaterium* WL-3 against *P. infestans* mycelium growth measured from the LC, CS, and CFS preparations showed average zones of inhibition 8.4, 12.3, and 9.5 mm, respectively, and average inhibition rates 75.0, 84.6, and 73.3%, respectively (Figure 1). The inhibitory effects of all three preparations were significantly different from that of the control (*P* < 0.05), and the inhibitory effect of CS was significantly greater than that of LC and CFS (*P* < 0.05). CS had no side effects on the tissues *in vitro*, and the treated tubers and leaflets appeared fresh and without deterioration (Supplementary Figure S1A and Table 1). CS inhibitory activity across all three infection/treatment scenarios, DP, SI, and DT, indicated a significant biocontrol effect on late blight (Supplementary Figures S1B–D and Table 1). On tubers *in vitro*, average DIs were 9.6, 17.1, and 38.4, respectively, and on leaflets *in vitro*, average DIs were 7.2, 14.4, and 32.7, respectively, all significantly lower than that of the corresponding control (*P* < 0.05) (Supplementary Figure S1E and Table 1).

**TABLE 1:** Biocontrol effect of *B. megaterium* WL-3 on potato tissues *in vitro.*

	Disease index (DI)	
	
Treatments	Tubers	Leaflets
CS only	0.00.00^a^	0.00.00^a^
Disease Prevention (DP)	9.60.45^b^	7.20.37^b^
Simultaneous inoculation (SI)	17.10.88^c^	14.40.70^c^
Disease Therapy (DT)	38.41.90^d^	32.71.69^d^
Control	72.23.66^e^	70.93.51^e^

*CS only, only using CS without infection; Control: LB liquid medium. Different letters a–e in the same column indicate a significant difference in DI between infection/treatment condition (*P* < 0.05). Data are expressed as the average of three replicates (total 36 tubers/leaflets per treatment) ± standard deviation.*

### Characterization of *B. megaterium* WL-3 CLE Components by MS Analysis

**FIGURE 2:**
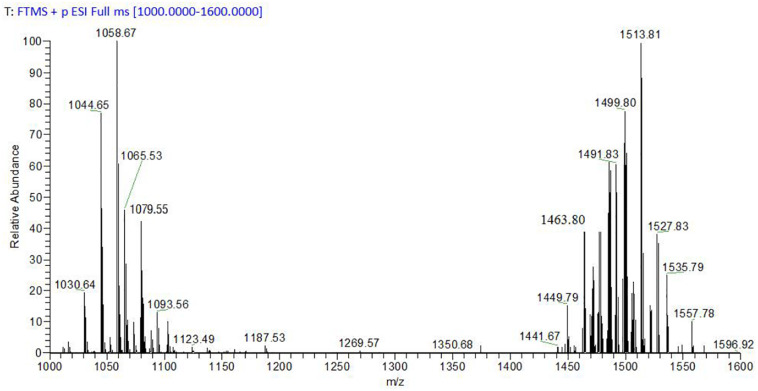
MALDI-TOF-MS detection of *B. megaterium* WL-3 CLE.

The average yield of prepared CLE was 2.8 g/L, and the average zone of inhibition for CLE (1 mg/mL) against *P. infestans* mycelium growth was 7.6 mm (Supplementary Figure S2). MS analysis was used to clarify the classification in *B. megaterium* WL-3 CLE (Figure 2). MS results exhibited peaks in the *m/z* ranging from 1,000 to 1,100 such as 1,030.64, 1,044.65, and 1,058.67 characteristic of the surfactin family with Na^+^ adduct ions bound to the C_13_ to C_15_ of the fatty acid chain (Figure 2). Signals at *m/z* 1,065.53, 1,079.55 and 1,093.56 were predicted to be Iturin A with Na^+^ adduct ions bound to the C_14_ to C_16_ fatty acid chain (Figure 2). Also, *m/z* ranging from 1,400 to 1,600, specifically the ion peaks at 1,449.79, 1,463.80, and 1,491.83, were considered to be representative of Fengycin A (C_15_, C_16_, and C_18_) or Fengycin B (C_13_, C_14_, and C_16_) with H^+^ adduct ions bound. The ion peaks at *m/z* 1,499.80, 1,513.81, and 1,527.83 were suspected to be either Fengycin A (C_17_ – C_19_) or Fengycin B (C_15_ – C_17_) with Na^+^ adduct ions bound (Figure 2).

### Purification and Identification of *B. megaterium* WL-3 CLPs

CLE components were purified using HPLC. Three obvious peaks occurred at 22.3 min (peak a), 24.4 min (peak b), and 25.7 min (peak c) (Supplementary Figure S3) and were collected for further identification using MS/MS system (Supplementary Figure S4). MS/MS analysis revealed that peak a contained the ion peaks at *m*/*z* 1,008.66 (C_13_), 1,022.67 (C_14_), and 1,036.69 (C_15_) bound with H^+^ adduct ions represented the surfactin (C_13_ - C_15_) subfamily (Supplementary Figure S4A and Table 2). The same secondary ion fragments appeared in 1,008.66 (Supplementary Figure S4B), 1,022.67 (Supplementary Figure S4C), and 1,036.69 (Supplementary Figure S4D) including 132 → 245 (227, −H_2_O) → 360 → 441 (−H_2_O) → 572 (554, −H_2_O) → 685 indicated the peptide sequence of Leu → Leu → Asp → Val → Leu → Leu. Also, the peaks from b^+^ ions at *m*/*z* 342 → 455 → 568 → 667 → 764 (−H_2_O) → 895 signified the connection form of β-OH fatty acid (C_13_) − Glu → Leu → Leu → Val → Asp → Leu → Leu (Supplementary Figure S4B). Differing by 14 Da (− CH_2_ −), the obvious ion peaks at *m*/*z* 356 (Supplementary Figure S4C) and 352 (−H_2_O, Supplementary Figure S4D) represent the end of the C terminus (β-OH fatty acid - Glu) of 1,022.67, and 1,036.69, respectively. The parent ion peaks from MS/MS results, specifically, 1,065.53 (C_14_), 1,079.55 (C_15_), and 1,093.56 (C_16_) represented the Iturin A family (peak b) with Na^+^ adduct ions bound and differed in C terminus (Supplementary Figure S4E and Table 2). The secondary ion peaks of 1,065.53 in Supplementary Figure S4F showed that the series of y^+^ ions at *m*/*z* 362 → 431 (−H_2_O) → 563 → 788 exhibited the fragment sequence of Asn −β–OH fatty acid (C_14_) → Ser → Asn → Pro – Gln. Detected from the b^+^ part, 726 → 639 → 525 → 428 → 300 represented the fragments loss of Ser, Asn, Pro, and Gln, respectively, as well as the final N terminus of Tyr – Asn was inferred by the final b^+^ ion of 300. Starting from y^+^ fragments, the ions at *m/z* 300 → 414 → 653 → 836 (−H_2_O) indicated the fragments connection sequence of Asn − Tyr → Asn →β–OH fatty acid (C_15_) → Ser – Asn, respectively (1,079.55, Supplementary Figure S4G). In addition, from the series of b^+^ part, the ions at *m*/*z* 248 → 362 → 431 (−H_2_O) → 670 (−H_2_O) denoted the connected sequence of Gln → Pro → Asn → Ser →β-OH fatty acid (C_15_) (1,079.55, Supplementary Figure S4G). The same above, from the MS/MS results of 1,093.56 (Supplementary Figure S4H), the most significant ions from the part of y^+^ at *m*/*z* 414 → 667 and the b^+^ fragment at *m*/*z* 431 (−H_2_O) → 684 (−H_2_O) indicated a structure of β-OH fatty acid (C_16_) which differed by 14 Da (– CH_2_ –) with that of 1,079.55 (C_15_). The MS/MS results of peak c indicated the Fengycin A (C_15_ – C_19_) subfamily added with Na^+^ adduct ions like 1,471.76 (C_15_), 1,485.78 (C_16_), 1,499.79 (C_17_), 1,513.81 (C_18_), and 1,527.83 (C_19_), respectively (Supplementary Figures S4I–N and Table 2). The most characteristic ions from the part of y^+^ at *m*/*z* 966 → 1,080 symbolized the peptide fragment with the sequence Ile – Tyr – Gln – Pro – Ala – Glu – Thr – Tyr → Orn (Supplementary Figures S4J–N). From the series of b^+^ part, the ions at *m*/*z* 368 (C_15_, Supplementary Figure S4J), 382 (C_16_, Supplementary Figure S4K), 396 (C_17_, Supplementary Figure S4L), 410 (C_18_, Supplementary Figure S4M), and 424 (C_19_, Supplementary Figure S4N) represented the C terminus of the β–OH fatty acid connected with Glu and differed by14 Da (– CH_2_ –).

**TABLE 2:** MALDI-TOF-MS/MS detection of purified CLPs.

	Fatty acid chain	Molecular formula	Calculated (*m/z*)	
			
Lipopeptides			[M+H]^+^	[M+Na]^+^
Surfactin (peak a)	C_13_	C_51_H_89_N_7_O_13_	1,008.66	–
	C_14_	C_52_H_91_N_7_O_13_	1,022.67	–
	C_15_	C_53_H_93_N_7_O_13_	1,036.69	1,058.67
Iturin A (peak b)	C_14_	C_48_H_74_N_12_O_14_	1,043.55	1,065.53
	C_15_	C_49_H_76_N_12_O_14_	–	1,079.55
	C_16_	C_50_H_74_N_12_O_14_	–	1,093.56
Fengycin A (peak c)	C_15_	C_71_H_108_N_12_O_20_	1,449.78	1,471.76
	C_16_	C_72_H_110_N_12_O_20_	1,463.80	1,485.78
	C_17_	C_73_H_112_N_12_O_20_	–	1,499.79
	C_18_	C_74_H_114_N_12_O_20_	–	1,513.81
	C_19_	C_75_H_116_N_12_O_20_		1,527.83

### Inhibitory Effect of Iturin A and Fengycin A

**FIGURE 3:**
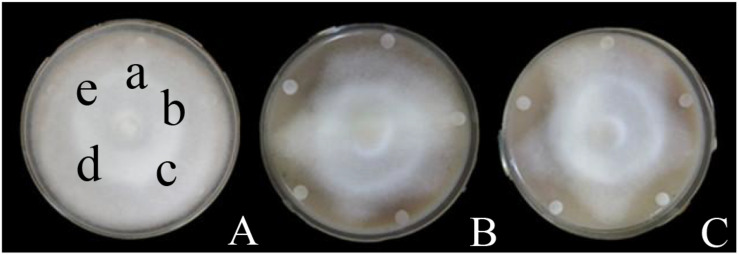
Inhibitory effects of surfactin, Iturin A, and Fengycin A on *P. infestans* mycelium growth. **(A)** Surfactin; **(B)** Iturin A; **(C)** Fengycin A. The letter a represents the control (distilled water), and the lower-case letters b-e indicate drugs concentrations of 10, 15, 20, and 25 μg/mL, respectively, the same in the **(B,C)** groups.

Disk diffusion assays were carried out to determine the inhibitory effects of the three purified CLPs on *P. infestans* mycelium growth (Figure 3 and Supplementary Table S1). Surfactin, even at the highest concentration (25 μg/mL), did not inhibit mycelium growth (Figure 3A and Supplementary Table S1), and there was no significant difference in zones of inhibition for the surfactin family from those of control (0.0 mm, *P* > 0.05) (Supplementary Table S1). While the inhibitory effects of Iturin A and Fengycin A increased in a dose-dependent manner (Supplementary Table S1 and Figures 3B,C). At the maximum concentration of 25 μg/mL, the zones of inhibition were 6.1 and 8.1 mm, respectively, and significantly different from that of control (*P* < 0.05) (Supplementary Table S1).

The ability of Iturin A and Fengycin A to control late blight was tested on infection-sensitive potato plant leaflets *in vitro* (Supplementary Figure S5). In the DP scenario, Iturin A and Fengycin A were each effective in controlling late blight, and the DIs were 22.2 and 14.5, respectively, both significantly lower than that of the control (77.9, *P* < 0.05) (Supplementary Figures S5A–C and Table 3). Similarly, in the DT scenario, the DIs for the Iturin A and Fengycin A groups were 32.6 and 25.7, respectively, also significantly lower than that of the control (76.1, *P* < 0.05) (Supplementary Figures S5A–C and Table 3). The I + F group exhibited an even more effective biocontrol activity against late blight on leaflets *in vitro* than either drug individually (Supplementary Figure S5D and Table 3), and in both DP and DT treatments, the DIs were only 6.2 and 10.1, respectively, significantly lower than that of the individual drugs in DP and DT (*P* < 0.05) (Table 3). In fact, the inhibitory effect of the I + F combination group in DP and DT treatments was close to that observed for the fungicide Metalaxyl-treated group which had DIs of 4.9 and 7.6, respectively (Supplementary Figure S5E). Furthermore, there was no significant difference between the I + F group and the Metalaxyl group (*P* > 0.05) (Table 3). Importantly, neither drug, Iturin A or Fengycin A, had side effects on the leaflets, and the treated leaflets appeared fresh and bright without any rotten spots (Supplementary Figure S5F).

**TABLE 3:** Biocontrol effect of Iturin A and Fengycin A on potato leaflets *in vitro*.

	Disease index (DI)	
	
Treatments	Disease prevention (DP)	Disease therapy (DT)
Control	77.93.99^a^	76.13.87^a^
Iturin A	22.21.09^b^	32.61.60^b^
Fengycin A	14.50.69^c^	25.71.33^c^
I + F	6.20.29^d^	10.10.49^d^
Metalaxyl	4.90.26^d^	7.60.36^d^

*Control (distilled water); I + F: Iturin A (25 μg/mL) + Fengycin A (25 μg/mL), 1:1 in volume; Metalaxyl (10 μg/mL), the same below. Data are presented as the average (total 36 leaflets per treatment) ± the standard deviation. Different letters a–d in the same column indicate a significant difference in DI between treatment groups (*P* < 0.05).*

### Greenhouse Experiments

#### Biocontrol Effect of Iturin A and Fengycin A on Potato Plants in Greenhouse Experiments

Greenhouse experiments were performed to assess the biocontrol effect of Iturin A and Fengycin A against *P. infestans* on potato plants (Table 4 and Supplementary Figure S6). The most serious DI occurred in the control group (sterile water), in which after 7 days of infection, the DI was 25.5, and by 28 days had reached 72.6, significantly higher at both timepoints than that of the Iturin A, Fengycin A, I + F, and Metalaxyl groups (*P* < 0.05) (Table 4). At 7 days after protection, the DIs of the Iturin A, Fengycin A, and I + F treatment groups were not significantly different from each other (*P* > 0.05); however, they were significantly different compared with the DI of the Metalaxyl group (*P* < 0.05). After treatment with either Iturin A or Fengycin A for 28 days, the DIs were only 46.2 and 27.8, respectively. At each recording time (days 14, 21, and 28), the DI for the Fengycin A group was significantly lower than that for the Iturin A group (*P* < 0.05), and the DI for the I + F group at 28 days of infection was 18.6, which was significantly lower than the DI when Fengycin A was applied alone (*P* < 0.05). In addition, the DIs calculated in I + F treatment had no significant difference from the DIs of the Metalaxyl group (days 14, 21, and 28, *P* > 0.05) (Table 4). The highest DRs in Iturin A, Fengycin A, and I + F groups appeared at 21 days after drug application, which were 30, 49, and 57%, respectively (Supplementary Figure S6). The DRs measured in the I + F group at days 14, 21, and 28 after drug application were significantly greater than the DRs in the individual Iturin A and Fengycin A groups (*P* < 0.05) yet were not significantly different from those of the Metalaxyl group at the same timepoints (*P* > 0.05) (Supplementary Figure S6).

**TABLE 4:** Disease index after Iturin A and Fengycin A protection in greenhouse experiments.

	Disease index (DI)				
	
Time (d)	Control	Iturin A	Fengycin A	I + F	Metalaxyl
7	25.51.23^a^	6.80.33^b^	4.50.21^b^	5.20.21^b^	1.10.04^c^
14	50.22.66^a^	24.21.17^b^	12.30.57^c^	6.00.27^d^	4.60.21^d^
21	67.13.41^a^	36.61.77^b^	17.90.81^c^	10.90.49^d^	12.50.59^d^
28	72.63.80^a^	46.22.34^b^	27.81.29^c^	18.60.88^d^	14.30.69^d^

*Data are presented as the average ± the standard deviation. Lower-case letters a–d indicate a significant difference between treatments within a timepoint (*P* < 0.05).*

#### Iturin A and Fengycin A Have Promotional Effects on Plant Growth and PPY in Greenhouse Experiments

Both Iturin A and Fengycin A had an obvious effect on promoting plant growth (Table 5). The average PHs of Iturin A and Fengycin A groups were 27.6 and 28.1 cm, respectively, significantly higher than that of the control (24.6 cm, *P* < 0.05) but not statistically different from the average PH of the I + F group (28.4, *P* > 0.05) (Table 5). Iturin A and Fengycin A each had a similar effect on the MSD with the average MSDs 11.4 and 12.3 mm, respectively, and both significantly higher than that of the control (9.3 mm, *P* < 0.05) (Table 5). Again, the I + F treatment was the most suitable method to promote stem growth with an average MSD of 13.2 mm which was significantly higher than that of either the Iturin A or the Fengycin A groups (*P* < 0.05) (Table 5). Regarding the MRN, Fengycin A and Iturin A groups counted the averages of 15.3 and 22.2 bars, respectively, both significantly higher than that of the control (13.1 bars, *P* < 0.05) (Table 5). However, the effect of single-drug application on promoting MRN was significantly lower than that of the I + F group, which was 27.3 bars (*P* < 0.05) (Table 5). Fengycin A and Iturin A treatments each had a great promotional effect on the PPY with the averages of 264.5 and 270.1 g/plant, respectively, both significantly higher than the average PPY of the control group (246.3 g/plant, *P* < 0.05) (Table 5). The combined I + F treatment resulted in the most remarkable improvement in PPY with an average of 281.3 g per plant (*P* < 0.05) (Table 5).

**TABLE 5:** Promotional effect of Iturin A and Fengycin A on potato plant growth.

	Treatments			
	
Measurements	Control	Iturin A	Fengycin A	I + F
PH (cm)	24.61.20^a^	27.61.35^b^	28.11.38^b^	28.41.33^bc^
MSD (mm)	9.30.44^a^	11.40.52^b^	12.30.62^b^	13.20.59^c^
MRN (bars)	13.10.58^a^	15.30.69^b^	22.21.06^c^	27.31.28^d^
PPY (g)	246.310.2^a^	264.512.2^b^	270.111.5^b^	281.313.1^c^

*PH (Plant height), MSD (Main stem diameter), MRN (Main root number), and PPY (Per plant yield). Data are presented as the average ± the standard deviation. Different letters a–d indicate a significant difference between treatments within a growth measurement (*P* < 0.05).*

### Field Trials

#### Biocontrol Effect of Iturin A and Fengycin A Against Late Blight in Field Trials

The biocontrol effects of Iturin A and Fengycin A individually and in combination were further studied in field trials (Table 6). The DI reached a maximum value of 72.2 at the fifth 10-day interval inspection of plants in the control group (Table 6). The most serious condition observed was the large-scale death of potato plants due to a late blight outbreak. The effect of late blight on the untreated plants was so serious that the DI could not be measured at the last two timepoints. However, the Fengycin A, Iturin A, and I + F groups were all significantly protected against late blight disease as measured by disease severity (Table 6). After Iturin A application, the DIs in the first four timepoints remained lower than 35, and the DI increased from the fifth timepoint onward reaching a maximum value of 55.8 at the seventh timepoint, which was still lower than that of the control (72.2, *P* < 0.05) (Table 6). With the application of Fengycin A, the DIs from the first five timepoints were <25, and the highest DI (39.5) appeared in the seventh timepoint, which was significantly lower than that of the control (72.2, *P* < 0.05) and significantly lower than that of Iturin A group as well (55.8, *P* < 0.05) (Table 6). The most effective lipopeptide applications for controlling late blight in the field was the combined treatment I + F. The DIs for the I + F group in the first five timepoints were all < 20, and the highest DI appeared in the seventh timepoint at only 26.6, which was significantly lower than that observed with either Fengycin A or Iturin A treatment alone (*P* < 0.05) (Table 6). Furthermore, there was no significant difference between the DI (the seventh timepoint) of the I + F group and that of the Metalaxyl group (23.9, *P >* 0.05) (Table 6). Individually, treatment with Fengycin A or Iturin A resulted in a slower disease progression compared with that of the control group, and the maximum DRs were 36.8 and 48%, respectively, both measured in the fourth timepoint (Supplementary Figure S7). The highest DR in this field study (54.9%) was measured in the I + F group and showed observable disease suppression at the fifth timepoint (Supplementary Figure S7). Also, from the second timepoint, the DRs in the I + F group were higher than that of either the Fengycin A or Iturin A alone groups (*P* < 0.05) (Supplementary Figure S7). In addition, the DRs of the I + F group were not significantly different from the DR of the Metalaxyl group for observations made after the fourth timepoint (*P >* 0.05) (Supplementary Figure S7). Taken together, these results indicate that the combined I + F treatment was the most effective measure to control potato late blight in the field trials.

**TABLE 6:** Disease index of potato late blight in field trials after CLPs protection.

	Disease index (DI)				
	
Times	Control	Iturin A	Fengycin A	I + F	Metalaxyl
1	29.21.39^a^	6.10.28^b^	7.60.31^b^	5.40.22^b^	6.90.29^b^
2	45.52.19^a^	17.30.81^b^	15.80.77^b^	10.60.49^bc^	4.60.20^c^
3	52.92.66^a^	20.60.99^b^	23.11.14^b^	12.90.55^c^	7.70.32^cd^
4	70.63.65^a^	33.81.58^b^	22.61.09^c^	19.70.91^c^	11.40.61^d^
5	72.23.59^a^	48.22.51^b^	24.21.11^c^	17.30.85^d^	19.20.99^cd^
6	–	51.92.60^b^	37.91.77^c^	25.11.32^d^	20.91.03^d^
7	–	55.82.82^b^	39.51.88^c^	26.61.34^d^	23.91.20^d^

*Times indicate recordings made at seven sequential 10-day intervals. In the control group (distilled water), the absence of data in the sixth and seventh timepoints is due to the late blight outbreak and subsequent large-scale plant death and the significant difference analysis in the sixth and seventh times is based on the fifth timepoint control results. Data are presented as the average ± the standard deviation. Different lower-case letters a–d indicate a significant difference between treatments within a timepoint (*P* < 0.05).*

#### Effect of Iturin A and Fengycin A on Photosynthetic Efficiency and Potato Yield

**FIGURE 4:**
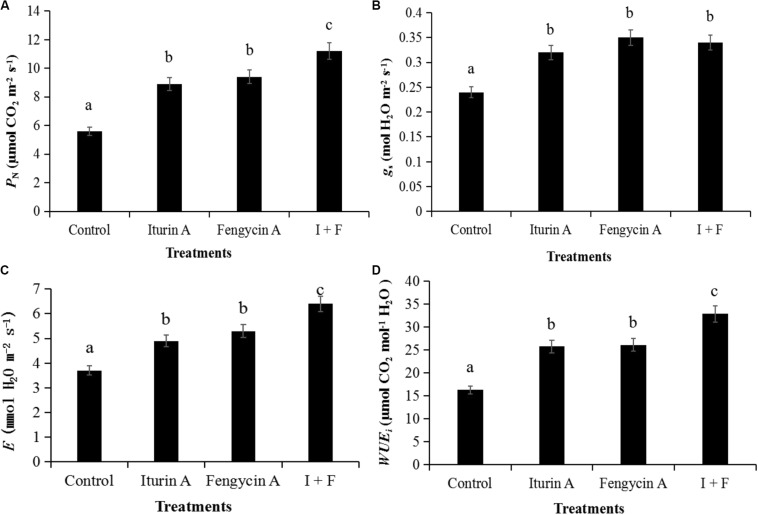
Parameters of photosynthetic efficiency in field trials. **(A)** Net photosynthetic rate (*P*_*N*_); **(B)** Stomatal conductance (*g*_*s*_); **(C)** Transpiration rate (*E*); **(D)** Intrinsic water use efficiency (*WUE*_*i*_). I + F, Iturin A (25 μg/mL) + Fengycin A (25 μg/mL), 1:1 in volume. Data are presented as the average ± the standard deviation, and the different letters a–c indicate a significant difference between treatment groups within a parameter (*P* < 0.05).

The application of Iturin A and Fengycin A in field trials significantly improved the photosynthetic efficiency at the aspects of *P*_*N*_, *g*_*s*_,*E*, and *WUE*_*i*_ of the potato plants (*P* < 0.05) (Figure 4). The promotional effect of I + F treatment on photosynthetic efficiency, as measured by the parameters *P*_*N*_, *E*, and *WUE_*i*_*, was significantly greater than that observed with the individual drug applications (*P* < 0.05) (Figures 4A,C,D). Using the parameter *g*_*s*_ as a measure of photosynthetic efficiency, the effect of treatment with either Fengycin A or Iturin A alone on *g*_*s*_ was not significantly different from the effect of the I + F group on *g*_*s*_ (*P* > 0.05); however, all treatment groups had a statistically greater *g*_*s*_ than that of the control group (*P* < 0.05) (Figure 4B).

The potato yield was evaluated in field trials, and the average potato number (per hill) of the Iturin A and Fengycin A groups were 6.1 and 6.4, respectively, both significantly higher than that of the control group (4.8 per hill, *P* < 0.05). The I + F treatment group was found to have the maximum potato number (7.8 per hill), which was significantly higher than that of the other treatments (*P* < 0.05) (Table 7). Besides, the average potato yield (per hill) of the Iturin A and Fengycin A groups were 712.3 and 798.5 g, respectively, both significantly higher than that of the control group (540.1 g, *P* < 0.05), but significantly lower than that of I + F group which had the maximum yield of 912.8 g per hill (*P* < 0.05) (Table 7).

**TABLE 7:** Promotional effect of Iturin A and Fengycin A on potato number and yield in field trials.

	Measurements	
	
Treatments	Number	Yield (g)
Control	4.8 ± 0.22^a^	540.1 ± 25.22^a^
Iturin A	6.1 ± 0.29^b^	712.3 ± 34.19^b^
Fengycin A	6.4 ± 0.30^b^	798.5 ± 38.81^c^
I + F	7.8 ± 0.38^c^	912.8 ± 46.72^d^

*Data are presented as the average ± the standard deviation, and different letters a–d indicate a significant difference between treatment groups (*P* < 0.05).*

## Discussion

Recently, the control of potato late blight has faced severe challenges due to the wide spread problem of the *P. infestans* A2 mating type (Wan, 2017) and the increased resistance of pathogenic strains caused by excessive use of chemical pesticides (Jin et al., 2017; Fukue et al., 2018). Additionally, the extensive use of chemicals to control late blight has greatly damaged the ecological environment and in itself poses a great threat to human health (Bajwa et al., 2003). In view of their adaptation to complex environments (Raaijmakers et al., 2010) and secretion of antagonistic metabolites, including proteins, lipids, polysaccharides, and peptides (Gao et al., 2017), the use of antagonistic *Bacillus* spp. (and their metabolites) to control plant diseases has attracted the attention of scientists engaged in the fight against these pathogens (Meena and Kanwar, 2015).

In this study, we identified the ability of *B. megaterium* WL-3 to inhibit *P. infestans* mycelium growth, and particularly the ability of *B. megaterium* WL-3 CS to inhibit the spread of mycelium on laboratory culture medium. However, with the variable influences and interactions present in the field environment, antagonistic activities that are obvious in sterile environments such as the lab are likely to be modified or completely missed (Ongena et al., 2005; Parthipan et al., 2017; Zihalirwa Kulimushi et al., 2017). The results of our study showed that the *B. megaterium* WL-3 strain has a strong ability to control late blight in plant tissues *in vitro*. A wide range of antimicrobial agents, especially CLPs secreted from *Bacillus* spp., such as surfactins, iturins, fengycins, bacillomycin, mersacidin, bacilysin, and subtilin, remain a focus of research in recent years (Rajesh Kumar et al., 2014; Wang, 2018). Here, CLPs belonging to *B. megaterium* WL-3 were characterized by MS/MS analysis, and three subfamilies, surfactin (C_13_ – C_15_), Iturin A (C_14_ – C_16_), and Fengycin A (C_15_ – C_19_) subfamilies, were found and proven to have the structures β–OH fatty acid – Glu – Leu – Leu – Val – Asp – Leu – Leu, β–OH fatty acid – Asn – Tyr – Asn – Gln – Pro – Asn – Ser, and β–OH fatty acid – Glu – Orn – Tyr – Thr – Glu – Ala – Pro – Gln – Tyr – Ile, respectively.

The surfactin family has been studied primarily for its antiviral, antibacterial, and antitumor properties (Meena and Kanwar, 2015; Zihalirwa Kulimushi et al., 2017). The iturins and fengycins families show prominent activities against a number of plant fungi pathogens (Zhang and Sun, 2018). As amphiphilic compounds, CLPs possess strong activities against a range of bacteria, viruses, and filamentous fungi through the disruption of cell membranes and intracellular structures (Ongena and Jacques, 2008; Zeriouh et al., 2014). However, the specific anti-oomycete activities of surfactins, iturins, and fengycins, especially those against *P. infestans* mycelium, are poorly understood. In this study, we found that the surfactin family had no inhibitory effect on the oomycete *P. infestans* mycelium growth. However, depending on the concentration, Iturin A and Fengycin A both exhibited obvious inhibitory effects against *P. infestans* mycelium growth. Additionally, the distinct antagonistic activities of CLPs subfamilies may be derived from their structural properties, such as the amino acid composition and sequence in the cyclic peptide (Wise et al., 2014), and the killing mechanism of CLPs is to target the ergosterol content in the pathogen cell membranes (Wise et al., 2014).

Synergistic effect between different CLPs against plant pathogens is still a controversial issue (Gong et al., 2015). For example, one report has claimed that CLPs extracted from *Bacillus amyloliquefaciens* FZB42 did not exhibit positive synergistic cooperation with surfactin, but the opposite was true since when *B. amyloliquefaciens* FZB42 fengycins were administered together with surfactin, the inhibitory effect of fengycins against *Rhizomucor variabilis* hyphal growth was weakened significantly (Zihalirwa Kulimushi et al., 2017). Additionally, when a mixture of surfactin and fengycins was used, the inhibitory effect of fengycins on *Verticillium dahlia* and *Rhizopus stolonifer* spore germination and hyphal growth was lost (Tao et al., 2011; Liu et al., 2014). In contrast, Gong et al. (2015) reported that surfactin and fengycins extracted from *B. amyloliquefaciens* JCK-12 could work as synergistic factors to promote the inhibitory effect of iturins against *F. graminearum* spore germination. Additionally, Kobayashi et al. (2002) reported that the cooperation of surfactin and fengycins significantly improved the antagonistic effects of CLPs against *Ralstonia solanacearum* and *Fusarium oxysporum* hyphal growth.

In this article, we have demonstrated that Iturin A and Fengycin A, working individually, had prominent controlling effects on late blight in *in vitro* experiments as well as in greenhouse experiments. Additionally, the synergistic antagonistic effect of Iturin A combined with Fengycin A (I + F) exerted significantly greater control than each CLP alone (*P* < 0.05). CLPs are versatile antimicrobial agents, but their antagonistic abilities are not easily presented in changing external environments (Glare et al., 2012). Our field tests showed that under the naturally variable environmental conditions, large-scale outbreaks of late blight were avoided with the protection of Fengycin A and Iturin A, and serious disease was controlled in the late stages of plant development. The most effective preventive measure was afforded by the combination of Fengycin A and Iturin A (I + F). With this combined treatment, even in the potato mature period, the DIs remained under 30. The synergistic effect of I + F may be because of drug differences and/or complementation between their inhibitory mechanisms (Kumar et al., 2012; Gu et al., 2017; Zhang and Sun, 2018). As demonstrated by Wise et al. (2014), the specific content of ergosterol in pathogen membranes determines their unique sensitivity to CLP drugs. Iturin A and Fengycin A (I + F) working together may target the most vulnerable sensitivities in the *P. infestans* membranes.

In the natural environment, many *Bacillus* spp. secrete CLPs capable of stimulating ISR that is dependent on the ethylene (ET) pathway in plants allowing the avoidance of disease and the adequate accumulation of nutrients (Ongena et al., 2007; Caulier et al., 2018). For example, *Bacillus mycoides* 15A-B2 was effective in controlling late blight severity by promoting potato plant growth (Caulier et al., 2018). Additionally, *B. amyloliquefaciens* VB7 and its CLPs promoted the growth of carnation stem cuttings, thus increasing flower yield (Vinodkumar et al., 2017). Similarly, our studies show that Iturin A and Fengycin A, either alone or in combination, had the exquisite ability to control late blight severity in greenhouse experiments as a result of promoting potato plant growth which was measured by the parameters PH, MSD, MRN, and PPY. Furthermore, the growth-promoting effect in the I + F application group was the most pronounced (*P* < 0.05). CLPs have attracted much attention because of their abilities to cause ISR in plants via salicylic acid (SA)- and jasmonic acid (JA)/ET-dependent pathways (Van Loon and Bakker, 2006; Saravanakumar et al., 2007; Klosterman et al., 2011). Chen (2017) reported that the SA, JA, and ET pathways could increase the *P*_*N*_, *g*_*s*_, and *E* significantly to improve organic matter accumulation (Wang et al., 2003; Chen, 2017). Sun et al. (2000) suggested that SA pathways could also increase the organic matter content per unit area of cucumber leaves. Similarly, in this study, we have demonstrated that Iturin A and Fengycin A, either alone or in combination, could improve the plant photosynthetic efficiency, as measured by *P*_*N*_, *E*, *g*_*s*_, and *WUE*_*i*_. In addition, the I + F combination was the best measure to ensure an improvement, and after I + F application, the increased photosynthetic efficiency combined with healthy growth without disease infection also increased potato yield in the field.

Taken together, this research demonstrates that *B. megaterium* WL-3, and specifically, the combination of its CLPs Iturin A and Fengycin A (I + F), have great potential in the goal to control potato late blight and to promote potato plant growth in an ever-changing and complex field environment. However, the possible anti-oomycete mechanisms of these two CLPs (Iturin A and Fengycin A), such as cell membrane leakage, organelle damage, ROS reaction, and DNA disruption, have yet to be investigated, and the indirect ISR involved in signal transduction and/or genes expression remains an open question that needs to be explored.

## Data Availability Statement

All datasets generated for this study are included in the article/Supplementary Material.

## Author Contributions

YW and JJ contributed to the conception and design of the study. YW, LW, and CZ performed all of the experimental work. JL and WG conducted the statistical analysis of experimental data. YW wrote the first draft of the manuscript. All the authors contributed to the manuscript revision as well as read and approved the submitted version.

## Conflict of Interest

The authors declare that the research was conducted in the absence of any commercial or financial relationships that could be construed as a potential conflict of interest.
