# Supplementary material for: Bacillus megaterium WL-3 Lipopeptides Collaborate Against Phytophthora infestans to Control Potato Late Blight and Promote Potato Plant Growth
Source: Front Microbiol. 2020 Jul 9;11:1602. doi: 10.3389/fmicb.2020.01602 (PMC7363778; doi:10.3389/fmicb.2020.01602)
Supplement: Supplementary file 1 [file Data_Sheet_1.doc]

**Supplementary Material**

## Supplementary Figures


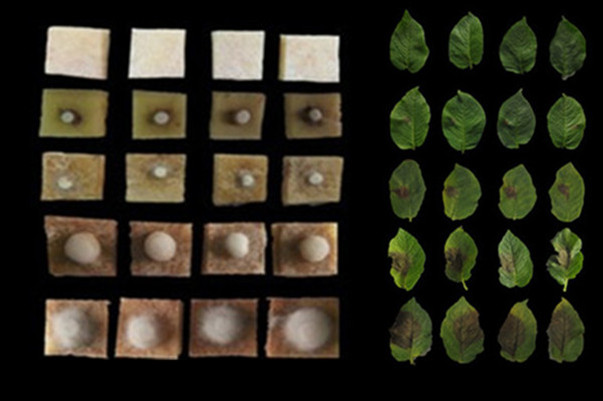


A

B

C

D

E

Tubers

Leaves

**Figure S1.** Biocontrol effect of *B. megaterium* WL-3 cell suspension (CS) on tubers and leaves *in vitro*. A: Only using CS without infection; B: Disease prevention (DP); C: Simultaneous inoculation (SI); D: Disease therapy (DT); E: Control (LB liquid medium). Photographs are representative of experiments performed in triplicate, the same below.


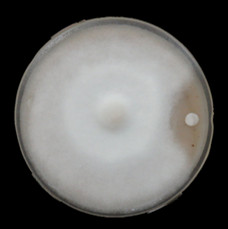


a

b

**Figure S2.** Inhibitory effect of crude lipopeptides extract (CLE) on mycelial growth. a: Control (distilled water); b: CLE (1 mg/mL).


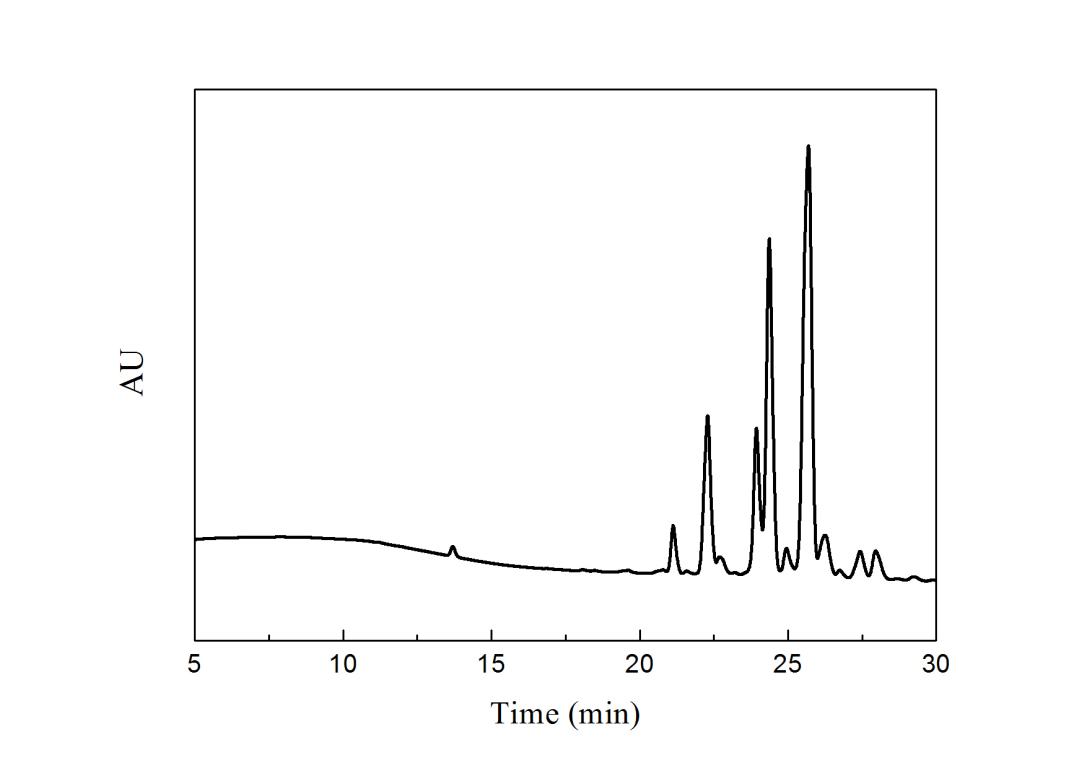


a

b

c

**Figure S3.** Elution profile for the separation of *B. megaterium* WL-3 CLE components using HPLC. Retention times of peaks a, b, and c were at 22.3 min, 24.4 min, and 25.7 min, respectively.


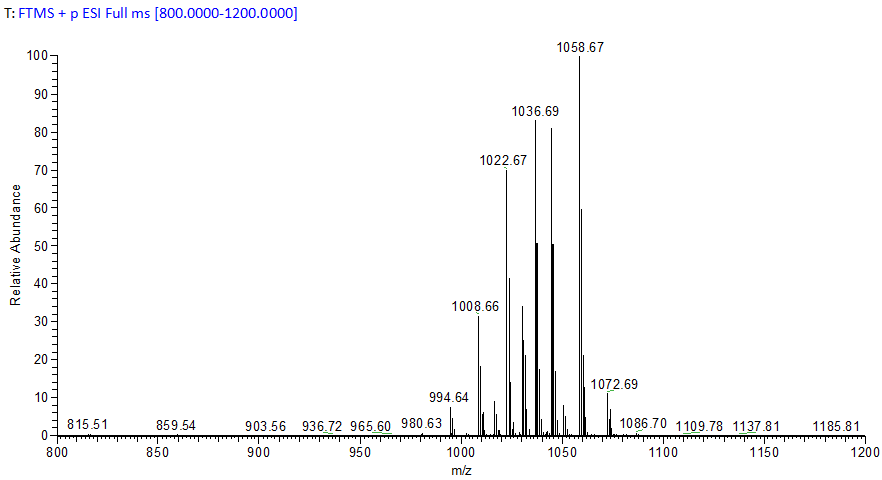

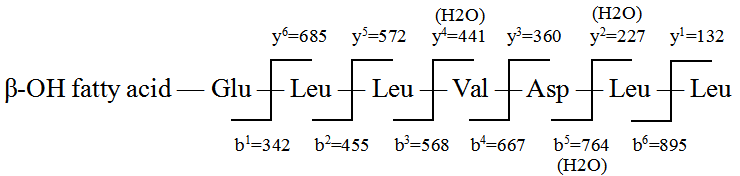

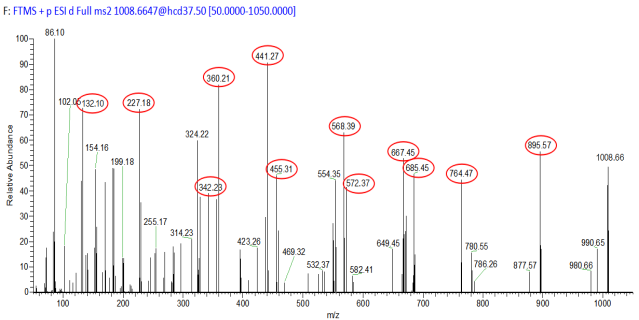

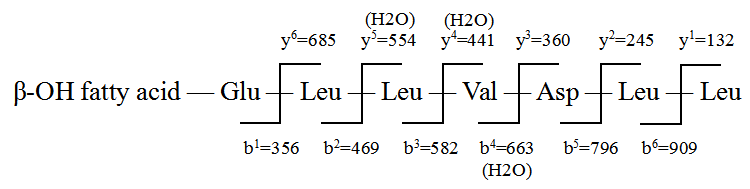

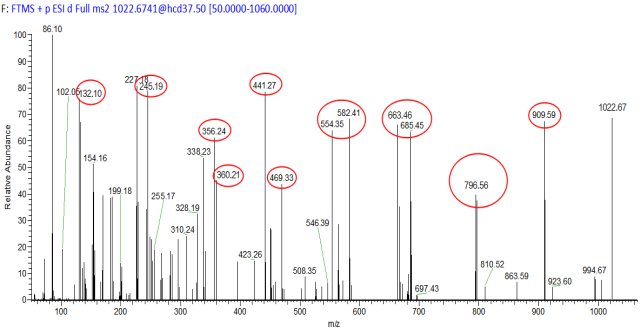

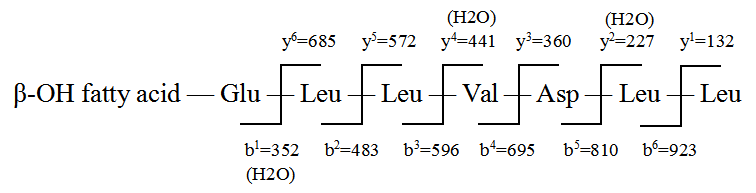

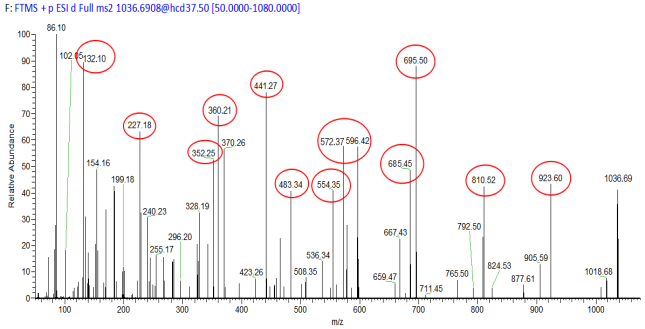


A

B

C

D


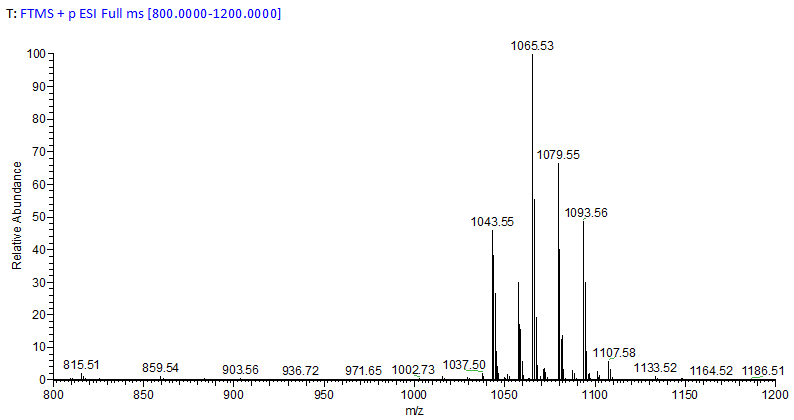

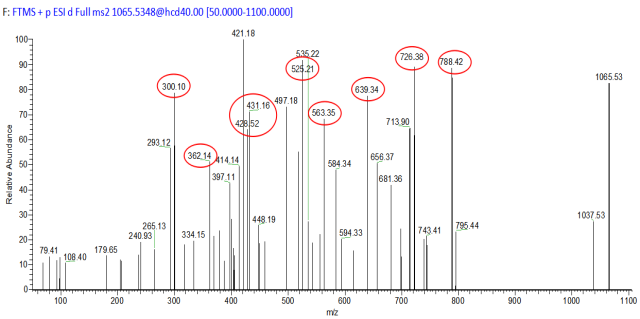

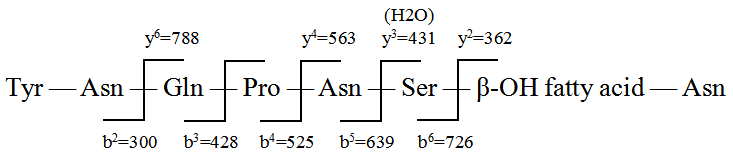


E

F


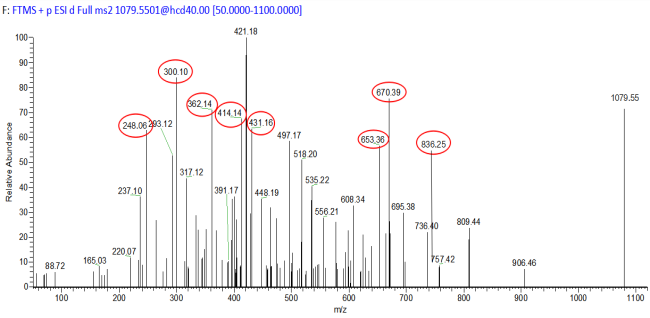

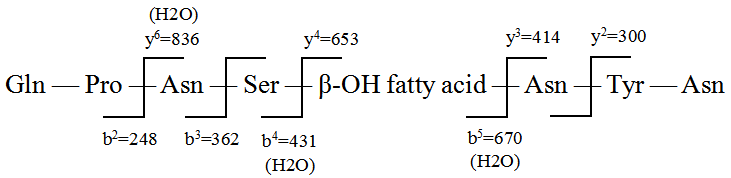

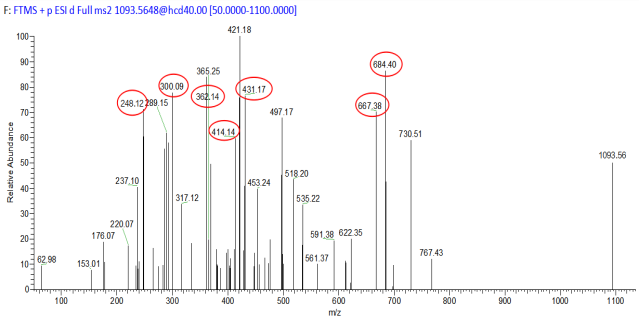

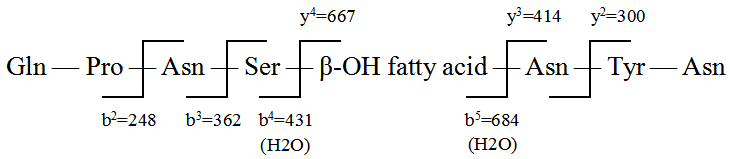


G

H


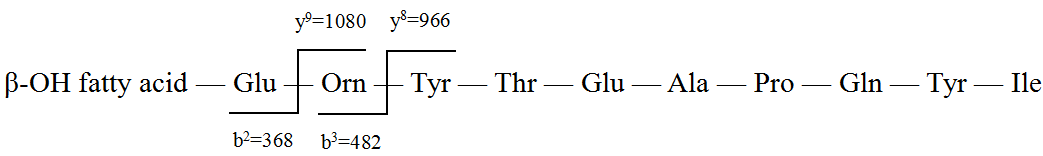

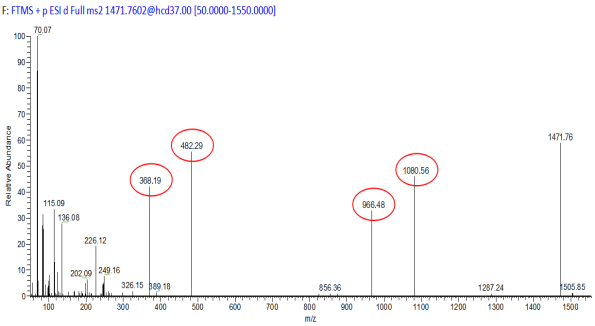

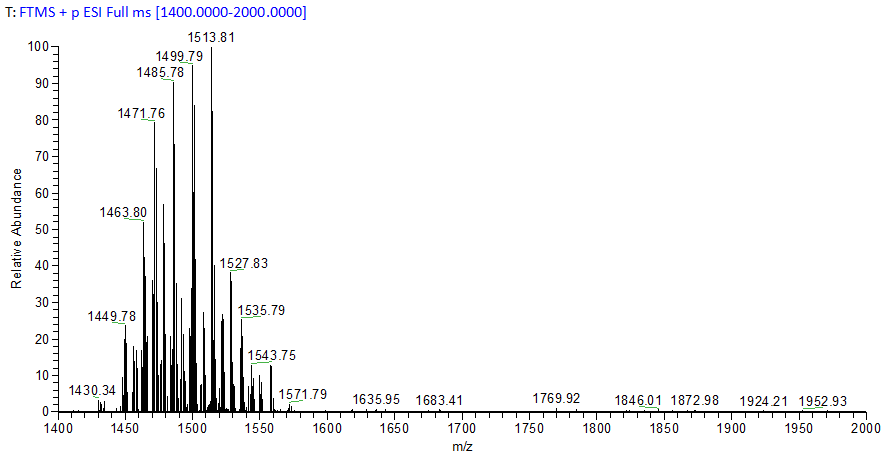

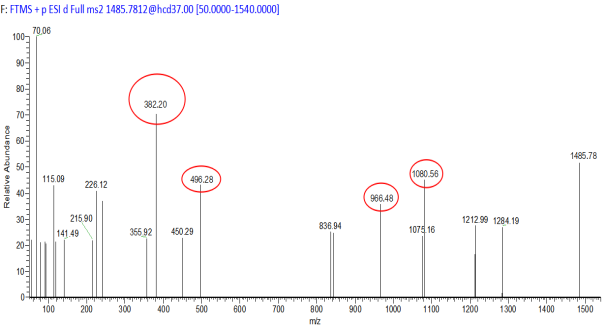

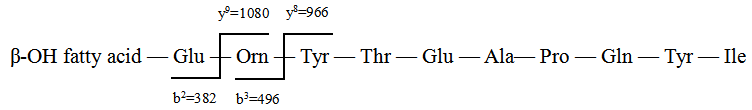

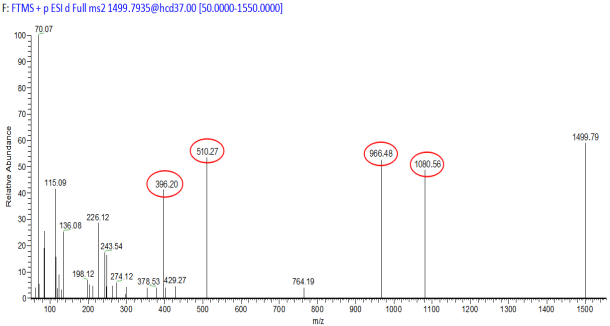

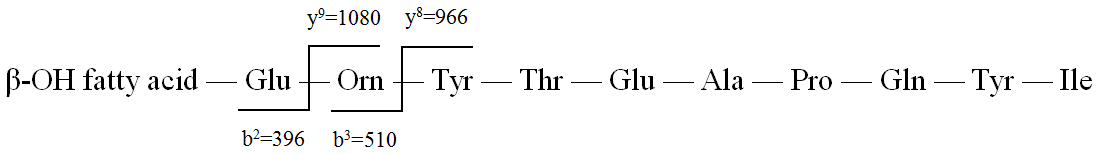

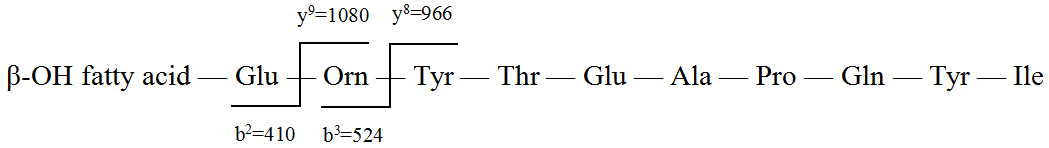

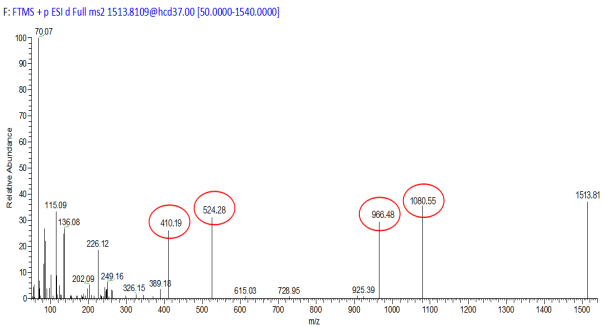

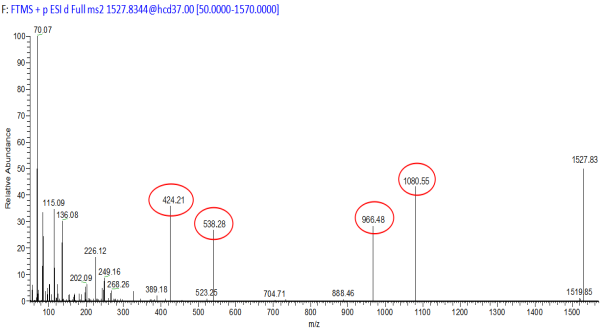

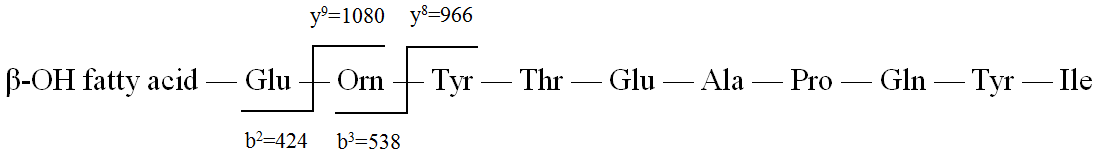


I

J

K

L

M

N

**Figure S4.** MS/MS analysis of purified CLPs. A, E, and I: Full mass spectra of peak a (surfactin), peak b (Iturin A), and peak c (Fengycin A), respectively. B-D: MS/MS spectra of the surfactin C_13_, C_14_, and C_15_ precursor ions at *m/z* 1,008.66, 1,022.67, and 1,036.69, respectively. F-H: MS/MS spectra of the Iturin A C_14_, C_15_, and C_16_ precursor ions at *m/z* 1,065.53, 1,079.55, and 1,093.56, respectively. J-N: MS/MS spectra of the Fengycin A C_15_, C_16_, C_17_, C_18_, and C_19_ precursor ions at *m/z* 1,471.76, 1,485.78, 1,499.79, 1,513.81, and 1,527.83, respectively. The important secondary ion fragments were marked using red circles.


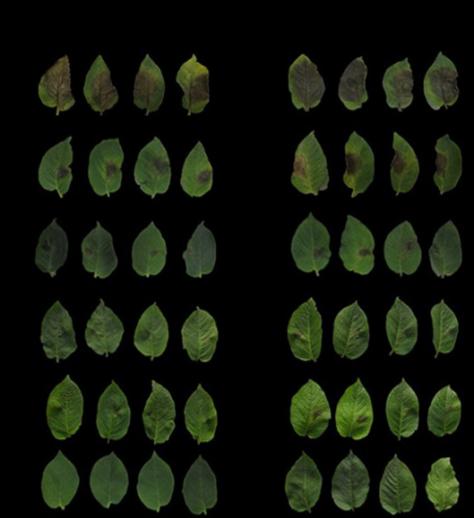


A

B

C

D

E

F

Prevention

Therapy

**Figure S5.** Biocontrol effect of Iturin A and Fengycin A on potato leaves *in vitro*. A: Control (distilled water), B: Iturin A (25 µg/mL), C: Fengycin A (25 µg/mL), D: I + F, Iturin A (25 µg/mL) + Fengycin A (25 µg/mL), 1:1 in volume, E: Metalaxyl (10 µg/mL), F: CLPs (I + F) were applied to the leaves in absence of *P. infestans* infection.

**
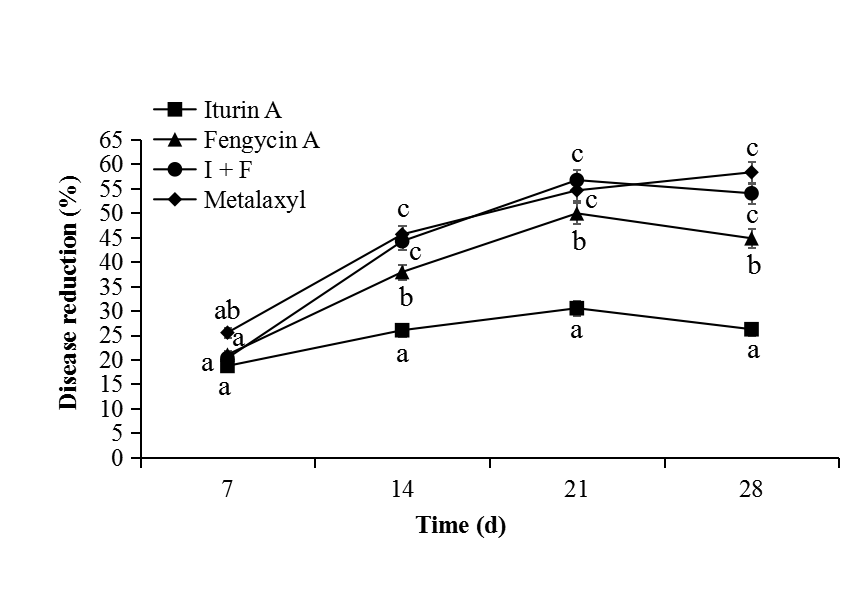
**

**Figure S6.** Disease reduction (DR) after treatment with Iturin A and Fengycin A in greenhouse experiments. Data are expressed as the average of three replicates ± standard deviation, and the lower-case letters a-c indicate a significant difference between different treatments within the same timepoint (*P*<0.05).

**
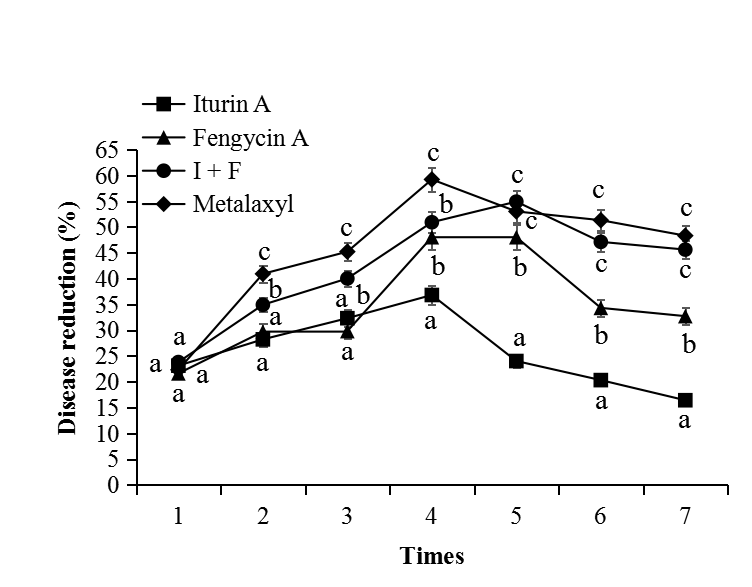
**

**Figure S7.** Disease reduction (DR) after application of Iturin A and Fengycin A in field trials. Times indicates timepoints at seven sequential 10-day intervals. Data are expressed as the average of three replicates ± standard deviation, and the lower-case letters a-c indicate a significant difference between treatments within the same timepoint (*P*<0.05).

**Supplementary Tables**

**Table S1.** Inhibitory effect of surfactin, Iturin A, and Fengycin A

| Concentrations(µg/mL) | Zones of Inhibition (mm) | | |
| --- | --- | --- | --- |
|  | Surfactin | Iturin A | Fengycin A |
| Control | 0.0 ± 0.00 a | 0.0 ± 0.00 a | 0.0 ± 0.00 a |
| 10 | 0.0 ± 0.00 a | 0.0 ± 0.00 a | 2.2 ± 0.08 b |
| 15 | 0.0 ± 0.00 a | 2.3 ± 0.09 b | 5.4 ± 0.24 c |
| 20 | 0.0 ± 0.00 a | 4.6 ± 0.18 c | 6.1 ± 0.29 cd |
| 25 | 0.0 ± 0.00 a | 6.1 ± 0.27 d | 8.1 ± 0.38 d |

Note: Data are expressed as the average of three replicates ± standard deviation, and the different letters a-d in the same column indicated a significant difference (*P*<0.05).
